# Development of Social Attention and Oxytocin Levels in Maltreated Children

**DOI:** 10.1038/s41598-020-64297-6

**Published:** 2020-05-04

**Authors:** Shizuka Suzuki, Takashi X. Fujisawa, Nobuko Sakakibara, Toru Fujioka, Shinichiro Takiguchi, Akemi Tomoda

**Affiliations:** 1 Division of Developmental Higher Brain Functions, United Graduate School of Child Development, University of Fukui, Fukui, Japan; 2 Department of Science of Human Development, Faculty of Education, Humanities and Social Sciences, University of Fukui, Fukui, Japan; 3 Department of Child and Adolescent Psychological Medicine, University of Fukui Hospital, Fukui, Japan; 4 Research Center for Child Mental Development, University of Fukui, Fukui, Japan

**Keywords:** Post-traumatic stress disorder, Paediatrics

## Abstract

Child maltreatment (CM) is a major risk factor for various psychopathologies but also adversely affects social development. Research on oxytocin (OT) is currently drawing attention as an endocrine basis for social development. In this study, we investigated the relationship between visual attention to social cues and salivary OT levels in children exposed to CM. The results revealed that the CM group had a significantly lower percentage of gaze fixation for the human face eye area and lower salivary OT levels compared to the typical development group. Moreover, a path analysis suggested that gaze fixation for the eye area was a mediator of the relationship between salivary OT levels and social-emotional problems in the CM group. These results suggest that lower endogenous OT levels in maltreated children may lead to atypical development of their visual attention to eyes as a social cue, resulting in social-emotional problems.

## Introduction

Child maltreatment (CM) includes physical abuse, sexual abuse, psychological abuse, and neglect, such as an inadequate caregiving environment or feeding, and is defined as “any act or series of acts of commission or omission by a parent or other caregiver that results in harm, potential for harm, or threat of harm to a child”^1^. This potential threat also includes exposure to domestic violence between parents. CM is not only a major risk factor for the development of psychopathologies, such as anxiety, substance abuse, psychosis, and personality disorders, but it is also associated with a host of neuropsychological and neurocognitive consequences^2^.

Moreover, previous studies have reported that CM adversely affects social development during childhood. Children with a history of CM were found to have significantly poorer skills in social interactions with peers and maintaining self-control, as well as various behavioral problems^3,4^. In addition, these deficits in social development domains resulting from CM have been shown to cause secondary problems, such as reduced self-esteem and depressive symptoms^5^, which can also adversely affect academic performance^6^. To take precautionary measures for maltreated children, CM needs to be detected as early as possible, creating a further need for a wider survey of related fields. CM is a global problem with adverse lifelong psychological and social functioning consequences; therefore, biomarkers and screening methods for early detection and intervention are necessary^2^.

Previous research has used eye-tracking technology to investigate visual attention patterns for early detection of atypical social development during childhood^7,8^. Gaze fixation duration for social stimuli, such as human faces or motions, predicts an individual’s ability to interpret others’ intentions and the meaning of social situations^9^. Thus, eye-tracking technology has several advantages for investigating visual attention during childhood^7^, allowing researchers to identify, with high precision and accuracy, what a participant is looking at and for how long. Moreover, eye-tracking technology applies to all populations, from infants to adults, irrespective of their verbal ability^10^. Therefore, different aspects of visual attention to social cues can be similarly investigated across various participant characteristics, such as age, gender, and clinical conditions such as autism spectrum disorder (ASD) have been researched^10^. Several studies have demonstrated that CM induces impaired social functions as well as atypical social development in conjunction with neuroendocrine dysfunction^11–15^. Moreover, recent research has reported that in children with CM, gaze fixation duration for emotional faces is significantly lower than that of children without CM^16^. Although it has often been suggested that children with neurodevelopmental disorders such as ASD or intellectual disability are at heightened risk for CM^17^, their commonality and heterogeneity in the atypical development of social attention have not been clarified, and the biological basis has not been researched.

Oxytocin (OT) signaling represents one of the most critical systems involved in human social behavior. OT, a neuropeptide secreted from the posterior pituitary, not only has physiological functions in labor and lactation, but it is also critical for post-natal mother-child bonding^18^. Recently, increasing evidence indicates that OT plays an important role in modulating social behavior in diverse species^19^. Previous human studies have suggested that OT administration facilitates sociability, such as inferring the mental state of others from viewing their eye region^20,21^, and that endogenous OT has a significant role in visual attention development to social cues during childhood^22^. OT level changes have also been suggested in the hormonal dysregulation associated with CM^12^. Previous studies have reported that women with a history of CM have lower OT concentrations in cerebrospinal fluid, and children who have experienced CM and lack attachment formation with a primary caregiver have atypical OT secretion patterns^13–15^.

In this study, we investigated the relationship between visual attention to social cues and salivary OT levels in children with a history of CM compared to children with TD. Although several studies have examined the relationship between CM and peripheral OT levels, or between CM and atypical social development, the association between the two in children with CM remains unclear. We measured visual attention patterns to social cues using eye-tracking, and salivary OT levels using enzyme-linked immunosorbent assays (ELISA), to investigate the relationships among these factors. Thus, our main hypothesis was that poor visual attention to social cues, and lower salivary OT levels would be observed in children with CM. In addition, we have previously reported that endogenous OT affects the development of individual differences in visual attention to social cues in healthy children^18^. Therefore, the subsidiary hypothesis is that the severity of developmental problems in CM is related to the atypical development of social attention based on endogenous OT dysfunction.

## Results

### Demographics, psychological, and behavioral assessments

We measured gaze fixation for various social cues and salivary OT levels and carried out several psychological and behavioral assessments in fifty children with or without exposure to CM. To eliminate the potential effects of age and gender, the groups were matched for these two factors^22–24^. The demographics, psychological, and behavioral characteristics of each group are listed in Table 1. First, we examined the differences between the groups (CM and TD) for the distribution of gender and the mean of their age using chi-square and t-tests, and confirmed that the two groups were matched for gender and age (*X*^2^(1) = 1.37, *p* = 0.44; *t*(48) = 1.37, *p* = 0.18). Next, when the differences between the groups were examined for the mean levels of the psychological and behavioral adaptation using the *t*-test, the CM group had significantly higher levels of adverse childhood experiences, social-emotional problems, and psychiatric symptoms compared to the TD group (ACE: *t*(48) = 12.92, *p* < 0.001; SDQ: *t* (48) = 3.49, *p* < 0.001; CMTI: *t*(48) = 5.75, *p* < 0.001)^27–29^. These results indicated that the CM group had lower adaptive psychological and behavioral function than the TD group.

**Table 1:** Participants’ demographic and psychological behavioral characteristics.

	CM (*n* = 21)	TD (*n* = 29)	Statistics	*P* value
Male participants, *n* (%)	11 (52.4)	12 (41.4)	*χ*^2^(1)=1.37	0.44
Age (years), *Mean* (SD)	5.5 (0.4)	4.8 (0.3)	*t*(48)=1.37	0.18
**Types of maltreatment**, ***n*** **(%)**				
Physical abuse	4 (19)	0 (0)	*χ*^2^(1)=6.0	0.14
Emotional abuse	10 (48)	0 (0)	*χ*^2^(1)=18	<0.001
Neglect	16 (76)	0 (0)	*χ*^2^(1)=32.49	<0.001
Sexual abuse	0 (0)	0 (0)	—	—
Duration (years) of maltreatment, *Mean* (*SD*)	3.2 (1.88)	0 (0)	*t*(48)=6.13	<0.001
Duration (years) elapsed from maltreatment, *Mean* (*SD*)	3.7 (1.84)	0 (0)	*t*(48)=10.8	<0.001
Duration (years) of residing in childcare facility, *Mean* (*SD*)	3.5 (0.47)	0 (0)	*t*(48)=8.80	<0.001
ACE total, *Mean* (*SD*)	3.95 (1.6)	0.07 (0.26)	*t*(48)=12.92	<0.001
SDQ total, *Mean* (SD)	11.48 (7.47)	5.9 (3.68)	*t*(48)=3.49	<0.001
CMTI, *Mean* (*SD*)	135.38 (29.65)	100.17 (12.50)	*t*(48)=5.75	<0.001

CM: Childhood Maltreatment, TD: Typical Development, ACE: Adverse Childhood Experience (Felitti *et al*., 1998), SDQ: Strength and Difficulties Questionnaire (Goodman *et al*., 2010), CMTI: Checklist for Maltreated Infant (Izumi *et al*., 2009).

### Between-group comparisons of gaze fixation

**Figure 1:**
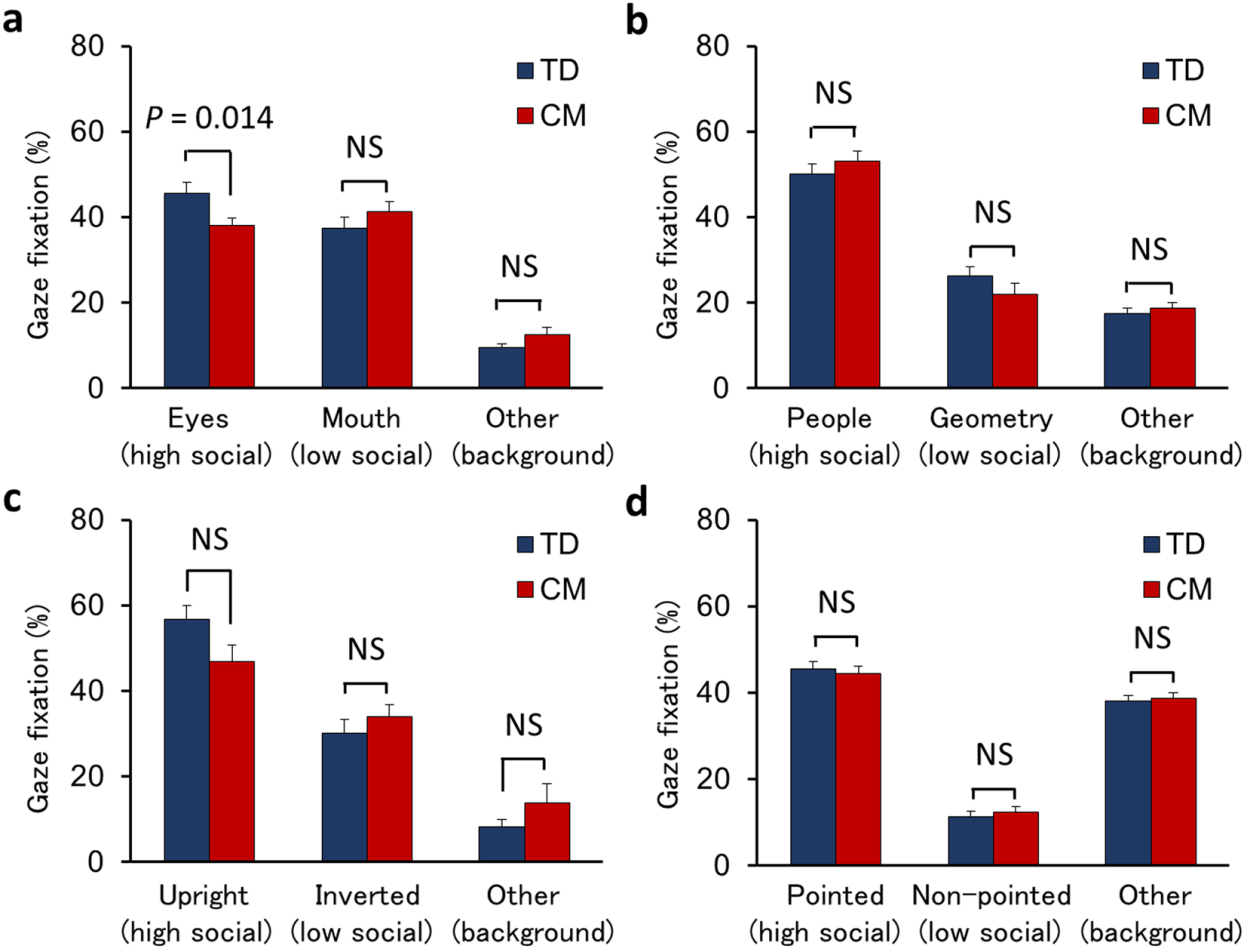
The percentage of gaze fixation duration of each AOI between the TD and CM groups: (**a**) Human face, (**b**) People and geometry, (**c**) Biological motion, and (**d**) Finger pointing. The vertical axis indicates the percentage of gaze fixation on each type of social cue. AOI, Area-of-Interest. TD, Typical Development. CM, Childhood Maltreatment.

The percentage of gaze fixation met the criteria of > 70% in all participants, with no significant differences between the groups on average (*t*(48) = −0.66, *p* = 0.511). Gaze fixation duration percentages for the four different types of social cues are presented in Fig. 1 (a: the human face, b: people and geometry, c: biological motion, and d: finger pointing; Snapshots of each type of stimulus can be found in ref. ^24^). Stimuli were presented as short movies, and each type of cue had three areas of interest (AOIs). The first AOI represented higher social cues with higher saliency (“high social”), the second AOI represented lower or non-social cues with higher saliency (“low social”), and the third AOI represented another area with lower saliency (“background”).

A two-way (2 × 3) analysis of variance (ANOVA) was performed to examine the differences in the percentage of gaze fixation between the groups (CM, TD) for each AOI. For human face stimuli, we did not observe a main effect of group (*F*(1, 48) = 0.09, *p* = 0.763), but a group × AOIs interaction (*F*(2, 96) = 3.16, *p* = 0.047). As a result of testing the simple main effect of the group for each AOI, a significant difference between the groups was confirmed only in high social (*F*(1, 144) = 6.25, *p* = 0.014), but not in low social and background (*F*(1, 144) = 1.54, *p* = 0.216; *F*(1, 144) = 1.04, *p* = 0.309). These results indicate that the fixation percentage on high social (eyes area) was significantly lower in the CM group than the TD group, but there was no significant difference in the fixation percentage for low social (mouth area) or background (other area) (Fig. 1a). On the other hand, for the other three types of social cues (people and geometry, biological motion, and finger pointing), we observed no main effects on the group for the fixation percentage (*F*(1, 48) < 0.01, *p* = 0.951; *F*(1, 48) = 0.10, *p* = 0.760; *F*(1, 48) = 0.06, *p* = 0.812, respectively) and no group × AOI interactions (*F*(2, 96) = 1.21, *p* = 0.302; *F*(2, 96) = 2.32, *p* = 0.103; *F*(2, 96) = 0.22, *p* = 0.800, respectively; Fig. 1b-d), although the main effects of AOIs confirmed that high social had a higher fixation percentage than low social and background (*F*(2, 96) = 105.92, *p* < 0.001; *F*(2, 96) = 52.90, *p* < 0.001; *F*(2, 96) = 191.85, *p* < 0.001, respectively). In addition, the effect of age on fixation percentage was not confirmed in high social AOIs, including human face stimuli, with observed differences between groups, and the effect was found to be limited in some low social and background AOIs (Table S1). These results indicate that the CM group does not demonstrate any atypical patterns in visual attention to social cues other than human faces.

### Between-group comparison of salivary OT levels

**Figure 2:**
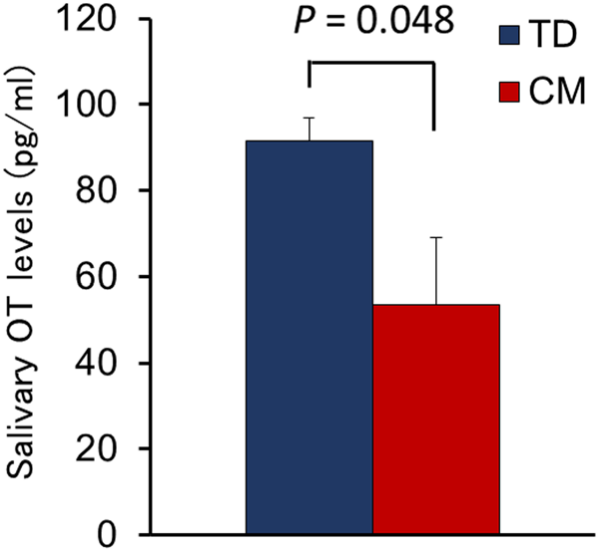
The percentage of salivary OT levels. OT, Oxytocin. TD, Typical Development. CM, Childhood Maltreatment.

To examine the relationship between endogenous OT levels and CM, we compared between-group salivary OT levels using independent *t*-tests and found that salivary OT levels were significantly lower in the CM group than the TD group (*t*(47) = −2.03, *p* = 0.048; Fig. 2).

### Relationship between gaze fixation and psychological,behavioral adaptation levels in the CM group

To investigate the relationship between atypical gaze fixation and psychological, behavioral adaptation levels in children with CM, we conducted a correlation analysis (Pearson’s *r*) with the psychological and behavioral scales for the high social area (eyes) in human face stimuli that showed a decrease in the percentage of gaze fixation in the CM group. The results indicated a significant negative correlation with the social-emotional problems (SDQ: *r* = −0.44, *p* = 0.048), but there was no correlation between the severity of adverse childhood experiences and psychiatric symptoms with exposure to CM (ACE: *r* = −0.24, *p* = 0.299; CMTI: *r* = −0.33, *p* = 0.139).

### Relationship between gaze fixation and salivary OT levels in the CM group

As described in the previous section, we observed a significant negative correlation between gaze fixation for the eye area in the human face (high social) and psychological and behavioral adaptation levels in the CM group. We then performed a correlation analysis for the CM group to confirm the relationship between gaze fixation and salivary OT levels, which demonstrated that salivary OT levels tended to correlate with percentage of fixation times for the eyes area in the human face, although the correlations did not reach a significant level (*r* = 0.42, *p* = 0.057).

**Figure 3:**
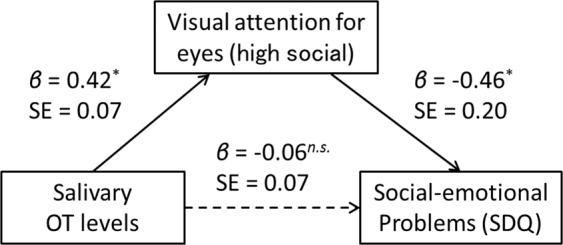
Path model for the mediation effect of visual attention on eyes in the relationship between salivary OT levels and social-emotional problems in maltreated children. OT, Oxytocin. AOI, Area-of-Interest. SDQ, Strength and Difficulties Questionnaire.

We also conducted a path analysis to examine whether gaze fixation for eyes mediated the relationship between salivary OT levels and social-emotional problems, despite no significant correlation in the direct relationship (*r* = −0.14, *p* = 0.334)^28,29^. As illustrated in Fig. 3, the salivary OT levels were a significant predictor of gaze fixation for eyes (*β* = 0.42, *t* = 2.08, *p* = 0.038). When salivary OT levels and gaze fixation for eyes were entered simultaneously as predictors of social-emotional problems (as measure by SDQ scores), the percentage of gaze fixation for eyes was a significant predictor (*β* = −0.46, *t* = −2.09, *p* = 0.037), while salivary OT levels were not (*β* = 0.06, *t* = 0.29, *p* = 0.775). The overall model excluding the path from OT levels to SDQ fit the data well (*X*^2^(1) = 0.08, *p* = 0.786, GFI = 0.997, AGFI = 0.984, RMSEA < 0.001, AIC = 10.081), while the indirect effect of salivary OT levels on social-emotional problems was estimated to remain moderate (*β* = 0.20). Thus, these findings suggest that visual attention to eyes mediates the relationship between endogenous OT levels in saliva and social-emotional problems,and the effect is restrictive.

## Discussion

We investigated the relationship between visual attention to social cues and salivary OT levels in children with CM and TD. The results revealed that the CM group had a significantly lower percentage of gaze fixation for the human face eye area compared to the TD group. We also found that salivary OT levels were lower in the CM group than the TD group. Moreover, the gaze fixation duration for the eye area in the CM group was negatively correlated with the degree of social-emotional problems and salivary OT levels. Accordingly, a path analysis suggested that gaze fixation for the eye area was a mediator of the relationship between salivary OT levels and social-emotional problems in the CM group. These results suggest that lower endogenous OT levels induced by exposure to CM may lead to atypical development of visual attention to eyes as a social cue, resulting in social-emotional problems in children with CM.

We found that visual attention to the eyes was lower in the CM group than in the TD group and that this was related to poor social-emotional functioning in the CM group. Previous studies have reported that children with CM had attention biases for human facial expressions compared to non-CM children, and that attention bias was associated with social-emotional problems^16,30^. Our results are consistent with these findings in that exposure to CM is associated with atypical development of visual attention to human faces, which is, in turn, associated with social-emotional problems in maltreated children. In contrast, other research has reported that children with disinhibited attachment disorder exposed to CM showed no attention bias to human faces compared with TD children^31^, suggesting that attention bias and social-emotional symptoms may change dynamically, depending on the type and timing of CM exposure^32,33^. On the other hand, lower visual attention in the CM group was not associated with the severity of adversity experience (ACE) or psychiatric symptoms associated with CM (trauma and sensory dysregulation; CMTI), suggesting that reduced social attention in the CM group is not directly related to the severity of CM exposure and associated psychiatric symptoms.

Typically, the gaze of others plays an important role as a social signal because it indicates the presence of a person or object that the person is paying attention to or interest in^34^. Therefore, studies have reported that people generally tend to pay attention to others’ eyes^35^. There are two possible explanations for why visual attention to eyes is lower in the CM group: “gaze avoidance” and “atypical distribution of attention.” According to the former explanation, it has been reported that people with social anxiety disorder avoid eye contact because of threats associated with gazing^36^, and CM experience has also been found to induce higher social anxiety^37^. Therefore, the high level of social anxiety induced by CM may lead to gaze avoidance. This notion is also largely consistent with the results of an earlier study that found that higher stress experiences in early childhood results in lower visual attention to facial expressions^38^. It is also possible that CM children’s visual attention tends to be directed to other areas (e.g., other face parts, situations) rather than the eye area.

Regarding the latter explanation, we also note that visual attention to social cues excluding eye area in human faces (people and geometry, biological motions, and finger pointing) were not significantly associated with CM exposure or OT levels in our current results. Previous studies targeting neurodevelopmental disorders, such as ASD in particular, have reported that the ASD group has a different distribution pattern of attentional allocation to social cues than the TD group^8,39^. It has been reported that children with ASD have reduced visual attention to social cues, such as human eyes, people, and biological motions^8,39^ while showing visual interest in inorganic cues such as geometric patterns^40^. Similarly, our previous study that presented the same stimuli also found that the ASD group had a lower gaze fixation for the human eye or people than the TD group, while fixation of the mouth or geometry area was higher^41^. However, the reduced visual attention of the CM group was specifically observed for the eye area in the current study. Taken together, the atypical visual attention of the CM group may be more rational to consider based on “gaze avoidance” rather than based on the “atypical distribution of attention” to general social cues as observed in the ASD group, which may involve endogenous OT dysfunction.

Many studies have reported endogenous OT dysfunction in maltreated children^19,21,23^. Fries *et al*. found that early neglected children had lower OT levels than control children after interaction with their mothers^21^. These results are consistent with the current study’s findings on the relationships between lower OT levels and childhood adversity, as represented by CM. Although several studies on the long-lasting effects of CM and effects on psychological health in adulthood, Heim *et al*. reported that adult women with a CM history had significantly lower OT levels extracted from cerebrospinal fluid compared to women without a CM history^13^. Similarly, Opacka-Juffry *et al*. also demonstrated that early childhood adverse experiences were negatively associated with plasma OT levels in adult men^42^. Although it should be noted that the findings from adulthood studies were compared with the results of the current study, the previous findings may be consistent with the association of CM exposure to reduce the endogenous OT levels in the CM group.

The gaze fixation analysis revealed a positive association between salivary OT levels and fixation for the human face eye area in the CM group. Several previous studies have suggested that OT exerts a positive effect on attention to social cues, including the eye region^20,21^. Similarly, impairments in social functioning, such as those seen in children with ASD, have been reported to be related to oxytocinergic system dysfunction^43^, and previous eye-tracking studies have revealed that children with ASD exhibit lower or altered visual attention to social cues^8,10^. Furthermore, our path analysis confirmed that reduced gaze fixation for the eye region mediated the relationship between salivary OT levels and social-emotional problems in maltreated children, although no direct relationship was found between the two. These findings suggest that endogenous OT is involved in the control of visual attention to social cues in maltreated children, particularly in regard to the human face eye area, and that lower OT levels indirectly affect social-emotional problems associated with atypical development in visual attention. This is the first study to elucidate the relationship between decreased social attention and social-emotional problems in maltreated children and to suggest that they may have dysfunctional endogenous OT.

This study has some limitations. First, the study had a relatively small sample size and used a cross-sectional design that precludes the identification of causal links between salivary OT levels, CM, and social-emotional problems. It was also difficult to consider potential impact differences related to the type or timing of CM. Longitudinal studies with larger sample sizes are necessary to better identify these associations. Second, the subsequent environmental factors after CM could not be controlled in this study because only children in residential childcare facilities were recruited as CM group participants. Similar investigations should be conducted with children in foster care. Third, none of the children in this study had a diagnosis of neurodevelopmental disorders, such as ASD in children aged 3 years or older, but the possibility could not be excluded in children under 2 years. In addition, as children in residential childcare facilities were sampled, it was difficult to obtain parental confirmation of the developmental difficulties evident before the children entered the facility. Longitudinal studies that compare CM children with innately high-risk ASD children are necessary.

Fourth, saliva sampling included only a single daytime measure. Although no significant diurnal variation in OT was confirmed, it is necessary to investigate potential variations over multiple time points^44^. Finally, the children’s cognitive abilities (IQ/DQ) and socioeconomic status (SES) were not controlled in group comparisons in this study. In this study, it was difficult to assess their cognitive abilities on a single scale because we studied children aged 2–9, in particular, to quantitatively assess the 12 children in the TD group using Denver II (Table S2). Therefore, the cognitive ability could not be analyzed as a covariate. Although both groups used IQ/DQ < 70 as an exclusion criterion, there were also substantial differences in cognitive abilities between the groups, as suggested in previous studies. In our previous study, using the same instrument for children with ASD, their reduced attention to social cues were not associated with IQ^41^. This finding may indicate that reduced social attention in the CM group in this study is not directly attributed to cognitive abilities. Regarding SES, it was difficult to obtain information on parents’ SES using a standardized scale, such as the Hollingshead two-factor index among children in residential care facilities. However, it was also confirmed in the current study that there was no significant difference in the distribution between groups in the questionnaire item about whether there were any financial difficulties in the past or present (*X*^2^(1) = 2.89, *p* = 0.089; Table S2). Despite these limitations, this study sheds light on the neurobehavioral underpinnings of social-emotional problems caused by an environmental factor, i.e., the adverse experiences of children with CM.

In conclusion, our results demonstrated significantly lower visual attention to eyes, a salient social cue, in the CM group than the TD group; and that salivary OT levels were lower in the CM group than the TD group. Moreover, salivary OT levels were positively related to visual attention to the eyes area in the CM group. These results suggest that CM exposure is associated with a reduction in endogenous OT, and that the reduction modulates atypical visual attention to the eyes in the CM group. In addition, the reduced social attention in the CM group was associated with the degree of social-emotional problems, and social attention was a mediator in the relationship between oxytocin and social-emotional problems. These results suggest that the development of social attention for eyes is modulated by endogenous OT, which may exert an effect on social-emotional problems in maltreated children. In the future, it is important to clarify the neural basis of visual attention to social cues by combining physiological indicators such as brain imaging.

## Methods

### Participants

Fifty-three Japanese children aged 2–9 years (25 boys, 28 girls; mean age ± SD: 5.2 ± 1.8 years) participated in this study. The maltreated group consisted of 21 children with maltreatment experiences (CM group) who were living in residential childcare facilities in Japan. All of the children in the CM group had a history of physical, sexual, or emotional abuse or neglect before coming to the facility. The control (TD) group consisted of 32 typically developing children with no history of maltreatment and who did not meet the DSM-5 criteria as assessed by administered licensed pediatric-psychiatric clinicians (SS, TF, and AT)^45^. The TD children were recruited from the local community. The children over 6 years had a full-scale intelligence quotient (IQ) > 70 on the Wechsler Intelligence Scale for Children-Fourth Edition or the Tanaka Binet Intelligence Scale-Fifth Edition (Japanese version of the Stanford-Binet Test), or a full-scale developmental intelligence quotient (DQ) > 70 on the Kyoto Scale of Psychological Development. The children under 5 years completed the Denver Developmental Screening Test (Denver II)^46–49^. Subsequently, three children who were assessed as having suspected or confirmed developmental delay were excluded. The protocol of this study was approved by the Ethics Committee of the University of Fukui (Assurance no. 20140142), and conducted following the Declaration of Helsinki. All parent(s) or childcare facility directors provided written informed consent for participation in this study.

### Gaze pattern measurement

We measured gaze pattern for children using Gazefinder® (JVC KENWOOD Corporation, Kanagawa, Japan), an eye-tracking system for responses to visual stimuli^22,24,41^. The apparatus recorded the percentage of fixation duration allocated to specific AOIs on a video monitor^22,24,41^. The Gazefinder® is equipped with infrared light sources and cameras with a 19-inch transistor monitor (1280 × 1024 pixels). Using corneal reflection techniques, the Gazefinder® records the X and Y coordinates of the eye position at a frequency of 50 Hz (i.e., 3000 data collections/minute). Stimuli presented by the Gazefinder® consisted of short movies, including four categories of social cues, which were (a) human faces, (b) people and geometric patterns, (c) the biological motion of a human, and (d) objects with or without finger pointing. First, the human faces of (a) are considered representative of social stimuli^50^, and humans appear to naturally pay attention to the face, especially to the eye area^22,51^. Next, the people and geometry of (b) were based on the preference paradigm movie and measures the degree of visual attention to social cues underlying the hypothesis that individuals with atypical development such as ASD tend to react to inorganic cues, such as geometric patterns relative to social stimuli^40^.

The biological motion movie of (c) also measures the degree of attention to social cues underlying the hypothesis that humans show an innate preference for the biological motion to facilitate adaptive interaction with others^52^. Finally, finger pointing of (d) is a significant part of the ability to establish join attention with others^53^, and various studies have reported a deficit in joint attention as one of the salient signs of atypical development^54^. Thus, based on the previous findings, the above four types of movies were presented as social cues by Gazefinder®. Three AOIs were set within each stimulus (Fig. 1a-d). The first AOI, high social, represented higher social cues with higher saliency (“eyes” in [a], “people” in [b], “upright figure” in [c], and “object with pointing” in [d]). The second AOI, low social, represented lower or non-social cues with higher saliency (“mouth” in [a], “geometry” in [b], “inverted figure” in [c], and “object without pointing” in [d]). The third AOI, background, represented the other areas with lower saliency^22,24,41^. Snapshots of the four types of stimuli presented can be found in the reference^24^.

### Procedure and stimuli

The experiments were conducted in a quiet room at the childcare facility for the CM group and at the university research laboratory for the TD group during the daytime from 9:00 to 17:00 (Table S2). The children were seated on a small chair, approximately 70 cm in front of the eye-tracking monitor. Parent or institution staff were instructed not to assist the children during the research procedure. To obtain calibration information, the children were instructed to look at the animated animal displayed in one of five locations on the monitor. If the calibration quality was poor on any of these points, the calibration process was repeated. Before the task, the children were informed that pictures of faces, people, and objects would be shown on the monitor, and they were instructed to look at them for as long as they could without looking away. Stimulus movies were organized in a definitive order, and the sequence was presented only once as a trial. Between the stimulus movies, an attention-catching animation with a voice saying “Hey! Look!” was presented in the center of the monitor to reorient the children’s attention to the stimuli^22,24,41^. The trial was repeated in case the percentage of gaze fixation for the screen did not meet the criteria of > 70%.

### Salivary OT levels measurement

We collected saliva samples using Salivettes® (Sarstedt, Rommelsdorft, Germany). Parents or facility staff were instructed to place the collected cotton in the children’s’ mouth and chew it for one minute until it was saturated with saliva^22,24^. This process was repeated to collect two cotton samples. The saliva samples were then stored frozen at −80 °C, and were lyophilized overnight at −20 °C to concentrate them 2- to 4-fold. Prior to the assay, the dry samples were reconstituted in the assay buffer using a commercial OT enzyme immunoassay kit (Enzo Life Sciences, Inc., NY, USA). These protocols were consistent with those of previous studies on adults^55–57^ as well as on 5- to 19-month-old children^22,24^. Each sample was examined in duplicate, and the concentrations were calculated using the microplate reader, according to relevant standard curves. The average intra-assay coefficients of variation (CV) was 8.2%.

### Psychological and behavioral characteristics assessment

In addition to the clinical assessments, we evaluated the participants’ psychological and behavioral characteristics using the following three scales under the supervision of pediatric psychology clinicians: the Adverse Childhood Experience (ACE) Questionnaire was used to assess the severity of ACEs, which addresses ten individual ACEs under three categories: abuse, neglect and household dysfunction (e.g., parental divorce, domestic violence)^25,58^. The ACE questionnaire is a reliable and valid measure of childhood adversity that has been used extensively in large-scale ACE studies^58^. In this study, we used a Japanese version of nine items that were modified for Japanese children^59^. The ACE score ranges from 0–9 and is the total number of childhood adversities (only one count per type of abuse) experienced before 18 years of age. Although no clear cut-off value was set in the ACE questionnaire, a recent review of the research suggests that participants who had ACE scores of 4 or more were at increased risk of all health outcomes compared to those who had no ACEs^60^. To assess the social-emotional problems, parents or caregivers at the residential childcare facility completed the Strength and Difficulties Questionnaire (SDQ) consisting of 25 items, which is widely used across different cultures and has confirmed reliability and validity in both the original and Japanese versions^61,62^. The SDQ is a simple adaptive functioning screen for children,and it is divided into five categories to assess children’s internalized (emotional and relationships) and externalized (conduct and hyperactivity) problems. Prosocial scores are excluded from the total scores as they are classified as a positive quality^26^. The items were scored on a 0–2 scale (not true, somewhat true, certainly true), and the total difficulty score is calculated by adding the results of the scales (excluding the prosocial behavior scale), which can range from 0 to 40. From the total difficulties score, the SDQ enables researchers to classify subjects as normal, borderline, or abnormal, based on cut-off points. In the Japanese version, 12 or less are assessed as “normal,” and 13 or more are assessed as “borderline” or “abnormal”^62^. To assess the severity of psychiatric symptoms observed in maltreated children in a residential childcare facility, parents or caregivers in residential childcare facility completed the Checklist for Maltreated Infant (CMTI) consisting of 81 items, including three subscales: trauma, attachment, and sensory regulation. The items were rated on a four-point Likert scale, ranging from 1 (not at all) to 4 (a very great degree), and the sum of the three subscales generates a total score, which can range from 81 to 324. The cut-off threshold for the total score is not defined. Reliability and validity analyses have proved CMTI to be an effective and practical tool^27^.

### Statistical analysis

We compared the group differences (CM and TD) between the demographics and clinical characteristics using chi-square and t-tests. To examine the effects of groups on each type of social cue (face, people, motion, and pointing), we computed the percentage of gaze fixation on the three AOIs and performed a two-way ANOVA with them as the dependent variable, and with AOIs (high social, low social, background) as the within-subjects variable and group (CM, TD) as the between group variable. In addition, the difference in salivary OT levels between groups was also examined using a t-test. Next, to examine the associations between the severity of clinical characteristics, atypical visual attention to social cues, and endogenous OT levels in the CM group, we calculated the correlation coefficients between their clinical scale scores, the percentage of gaze fixation in AOIs of social cues showing differences between groups, and the salivary OT levels. Finally, to test the validity of the model that OT dysfunction is involved in the adaptive functioning of the CM group via atypical social attention, we examined the relationship between the variables using a path analysis. The significance level was set to *p* < 0.05, and all of the statistical analyses were conducted using IBM SPSS Statistics and Amos for Windows, version20 (IBM Corp., Armonk, NY).

## Supplementary information

Supplementary Information.
