# Supplementary material for: Development of Social Attention and Oxytocin Levels in Maltreated Children
Source: Sci Rep. 2020 May 4;10:7407. doi: 10.1038/s41598-020-64297-6 (PMC7198561; doi:10.1038/s41598-020-64297-6)
Supplement: Supplementary file 1 — Supplementary Information. [file 41598_2020_64297_MOESM1_ESM.docx]

**Supplementary Information**

**Development of Social Attention and Oxytocin Levels in Maltreated Children**

Shizuka Suzuki^a^, Takashi X. Fujisawa^a,b^, Nobuko Sakakibara^a^, Toru Fujioka^c^,

Shinichiro Takiguchi^d^ and Akemi Tomoda^a,b,d,*^

^a^Division of Developmental Higher Brain Functions, United Graduate School of Child Development, University of Fukui, Fukui, Japan.

^b^Research Center for Child Mental Development, University of Fukui, Fukui, Japan.

^c^Department of Science of Human Development, School of Education, University of Fukui, Fukui, Japan.

^d^Department of Child and Adolescent Psychological Medicine, University of Fukui Hospital, Fukui, Japan.

*to whom correspondence should be addressed: [atomoda@u-fukui.ac.jp](file:///C:\Users\kodomo\Desktop\鈴木先生視線\論文\revised\toukou\atomoda@u-fukui.ac.jp)

**Supplementary Table S1.**

Multiple regression analysis on the effects of group and age for each AOI.

| **Dependent variable** | **Independent variable(s)** | | | | | |
| --- | --- | --- | --- | --- | --- | --- |
|  | **Group** | | | **Age** | | |
|  | *β* | *t* | *p* | *β* | *t* | *p* |
| *Human Face* |  |  |  |  |  |  |
| Eyes (high social) | -0.329 | -2.336 | **0.024** | 0.086 | 0.612 | 0.544 |
| Mouth (low social) | 0.189 | 1.317 | 0.194 | -0.217 | -1.509 | 0.138 |
| Other (background) | 0.211 | 1.507 | 0.139 | 0.223 | 1.590 | 0.119 |
| *People and geometry* |  |  |  |  |  |  |
| People (high social) | 0.184 | 1.296 | 0.201 | -0.283 | -1.995 | 0.052 |
| Geometry (low social) | -0.281 | -2.197 | **0.033** | 0.485 | 3.795 | **<0.001** |
| Other (background) | 0.159 | 1.127 | 0.266 | -0.304 | -2.152 | **0.037** |
| *Biological motion* |  |  |  |  |  |  |
| Upright (high social) | -0.255 | -1.799 | 0.078 | -0.120 | -0.843 | 0.403 |
| Inverted (low social) | 0.124 | 0.841 | 0.404 | -0.021 | -0.143 | 0.887 |
| Other (background) | 0.151 | 1.042 | 0.303 | 0.159 | 1.098 | 0.278 |
| *Finger pointing* |  |  |  |  |  |  |
| Pointed (high social) | -0.015 | -0.107 | 0.915 | -0.242 | -1.676 | 0.100 |
| Non-pointed (low social) | 0.039 | 0.268 | 0.790 | 0.224 | 1.547 | 0.129 |
| Other (background) | 0.108 | 0.761 | 0.451 | -0.299 | -2.106 | **0.041** |

**Supplementary Table S2.**

Demographic dataset of participants.

| **ID** | **Group (CM)** | **Age (months)** | **Gender** | **IQ** | **DQ** | **Scale (IQ/DQ)** | **Collection time** | **Financial difficulty** |
| --- | --- | --- | --- | --- | --- | --- | --- | --- |
| 1 | 1 | 24 | 1 |  | 96 | Kyoto scale | 9:12 | 1 |
| 2 | 1 | 33 | 1 |  | 89 | Kyoto scale | 9:24 | 0 |
| 3 | 1 | 41 | 0 |  | 78 | Kyoto scale | 9:31 | 1 |
| 4 | 1 | 50 | 1 |  | 73 | Kyoto scale | 9:38 | 0 |
| 5 | 1 | 78 | 1 | 74 |  | Tanaka-Binet V | 9:46 | 0 |
| 6 | 1 | 56 | 0 |  | 88 | Kyoto scale | 10:05 | 0 |
| 7 | 1 | 62 | 1 | 106 |  | WISC-IV | 10:12 | 0 |
| 8 | 1 | 66 | 0 |  | 104 | Kyoto scale | 10:19 | 0 |
| 9 | 1 | 66 | 1 |  | 94 | Kyoto scale | 10:25 | 0 |
| 10 | 1 | 67 | 1 | 103 |  | WISC-IV | 10:32 | 0 |
| 11 | 1 | 69 | 0 |  | 111 | Kyoto scale | 10:38 | 0 |
| 12 | 1 | 72 | 0 |  | 87 | Kyoto scale | 10:44 | 1 |
| 13 | 1 | 75 | 0 |  | 79 | Kyoto scale | 10:54 | 0 |
| 14 | 1 | 78 | 1 |  | 82 | Kyoto scale | 11:01 | 1 |
| 15 | 1 | 78 | 1 |  | 100 | Kyoto scale | 11:08 | 1 |
| 16 | 1 | 88 | 0 | 85 |  | WISC-IV | 11:15 | 0 |
| 17 | 1 | 92 | 0 |  | 91 | Kyoto scale | 11:21 | 0 |
| 18 | 1 | 105 | 0 | 91 |  | WISC-IV | 11:28 | 0 |
| 19 | 1 | 111 | 0 | 85 |  | WISC-IV | 11:34 | 0 |
| 20 | 1 | 54 | 0 |  | 85 | Kyoto scale | 11:44 | 0 |
| 21 | 1 | 24 | 1 |  | 86 | Kyoto scale | 10:45 | 0 |
| 22 | 0 | 83 | 1 | 124 |  | WISC-IV | 9:08 | 0 |
| 23 | 0 | 77 | 1 | 94 |  | WISC-IV | 9:12 | 0 |
| 24 | 0 | 57 | 0 | 110 |  | WISC-IV | 9:06 | 0 |
| 25 | 0 | 74 | 1 | 130 |  | WISC-IV | 11:22 | 0 |
| 26 | 0 | 45 | 0 | 100 |  | WISC-IV | 11:27 | 0 |
| 27 | 0 | 53 | 1 | 102 |  | WISC-IV | 15:08 | 0 |
| 28 | 0 | 51 | 0 | 109 |  | WISC-IV | 17:14 | 0 |
| 29 | 0 | 37 | 0 |  | >80 | Denver II | 14:47 | 0 |
| 30 | 0 | 95 | 0 | 102 |  | WISC-IV | 10:12 | 0 |
| 31 | 0 | 62 | 1 |  | >80 | Denver II | 10:50 | 0 |
| 32 | 0 | 63 | 0 | 90 |  | WISC-IV | 11:06 | 0 |
| 33 | 0 | 62 | 0 | 113 |  | WISC-IV | 12:56 | 0 |
| 34 | 0 | 44 | 1 |  | >80 | Denver II | 14:18 | 0 |
| 35 | 0 | 63 | 1 |  | >80 | Denver II | 15:18 | 0 |
| 36 | 0 | 24 | 1 |  | >80 | Denver II | 15:21 | 0 |
| 37 | 0 | 27 | 0 |  | >80 | Denver II | 14:16 | 0 |
| 38 | 0 | 73 | 0 | 90 |  | WISC-IV | 10:03 | 0 |
| 39 | 0 | 61 | 0 | 112 |  | WISC-IV | 11:08 | 0 |
| 40 | 0 | 65 | 1 | 98 |  | WISC-IV | 13:04 | 0 |
| 41 | 0 | 70 | 0 | 97 |  | WISC-IV | 14:12 | 0 |
| 42 | 0 | 67 | 1 | 101 |  | WISC-IV | 17:08 | 1 |
| 43 | 0 | 32 | 0 |  | >80 | Denver II | 11:03 | 0 |
| 44 | 0 | 80 | 1 |  | >80 | Denver II | 12:57 | 0 |
| 45 | 0 | 52 | 1 |  | >80 | Denver II | 13:01 | 0 |
| 46 | 0 | 27 | 1 |  | >80 | Denver II | 14:40 | 0 |
| 47 | 0 | 93 | 1 | 105 |  | WISC-IV | 14:56 | 0 |
| 48 | 0 | 61 | 1 | 109 |  | WISC-IV | 14:52 | 0 |
| 49 | 0 | 44 | 1 |  | >80 | Denver II | 10:05 | 0 |
| 50 | 0 | 36 | 1 |  | >80 | Denver II | 16:34 | 1 |

CM: Childhood Maltreatment, IQ: Intelligence Quotient, DQ: Developmental Quotient, Kyoto scale: Kyoto Scale of Psychological Development (Ikuzawa et al., 2002), Tanaka-Binet V: Tanaka-Binet Intelligence Scale V (Sugihara et al., 2003; Japanese version of the Stanford-Binet test), WISC-IV: Wechsler Intelligence Scale for Children-Fourth Edition (Wechsler, 2003), Denver II: Denver Developmental Screening Test–Revised (Frankenburg, 1998).
